# Vitamin K1 Exerts Antiproliferative Effects and Induces Apoptosis in Three Differently Graded Human Colon Cancer Cell Lines

**DOI:** 10.1155/2015/296721

**Published:** 2015-05-17

**Authors:** Antonella Orlando, Michele Linsalata, Valeria Tutino, Benedetta D'Attoma, Maria Notarnicola, Francesco Russo

**Affiliations:** ^1^Laboratory of Nutritional Pathophysiology, National Institute for Digestive Diseases IRCCS “Saverio de Bellis”, Castellana Grotte, 70013 Bari, Italy; ^2^Laboratory of Nutritional Biochemistry, National Institute for Digestive Diseases IRCCS “Saverio de Bellis”, Castellana Grotte, 70013 Bari, Italy

## Abstract

Vitamin K1 has been demonstrated as having anticancer potentiality mainly in liver cancer cells. Beyond the reported mechanisms of cancer inhibition (cell cycle arrest and induction of apoptosis), a possible control by vitamin K1 on molecules affecting cell growth could be hypothesized. In the literature, few (if any) data are available on its antitumor effects on colon cancer cells. Therefore, the aims of the study were to investigate in three differently graded human colon cancer cell lines (Caco-2, HT-29, and SW480) the effects of increasing concentrations of vitamin K1 (from 10 *μ*M to 200 *μ*M) administered up to 72 h on (1) cell proliferation, (2) apoptosis with the possible involvement of the MAPK pathway, and (3) polyamine biosynthesis. Vitamin K1 treatment caused a significant antiproliferative effect and induced apoptosis in all the cell lines, with the involvement of the MAPK pathway. A concomitant and significant decrease in the polyamine biosynthesis occurred. This is the first study demonstrating a significant polyamine decrease in addition to the antiproliferative and proapoptotic effects following vitamin K1 administration to colon cancer cell lines. Therapeutically, combinations of vitamin K1 with polyamine inhibitors and/or analogues may represent a suitable option for chemoprevention and/or treatment in future strategies for colorectal cancer management.

## 1. Introduction

Colorectal cancer (CRC) is a major health problem in industrialized countries [1], but the treatment is still far from being satisfying since approximately 90% of patients do not respond to chemotherapy protocols [2].

Thus, the development of new treatment modalities is necessary to improve the overall survival rate of patients with CRC.

In the last years, an emerging role has been attributed to micronutrients, such as vitamin K (VK). VK is an essential vitamin that was discovered as a fat-soluble antihemorrhagic agent. Physiologically, VK acts as cofactor for the *γ*-carboxylation of selected glutamates at the N-terminus of prothrombin and other VK-dependent coagulation factors. VK is a family of structurally similar 2-methyl-1,4-naphthoquinones, including phylloquinone (VK1), menaquinone (VK2), and menadione (VK3). All members of the VK family possess an identical naphthoquinone skeleton with various side chains that distinguish them. VK1 is found in many higher plants as well as algae, with the highest concentrations found in green leafy vegetables. VK2 also occurs naturally but is not made by plants being produced by a wide array of bacteria in the intestine. VK3 is not considered a natural VK, but rather a synthetic analogue acting as a provitamin with a much simpler structure and no aliphatic chain prosthetic group at position 3 [3].

Although VK is usually identified as a critical factor in blood coagulation, recent research has demonstrated its ability to inhibit cancer cell growth in both* in vitro* and* in vivo* studies [4]. The naturally occurring VK and its analogs are able to inhibit the survival of various cancer cell lines [5, 6]. Among the various mechanisms suggested as explaining these effects, the altered expression of some growth related genes (e.g., cyclin D1, cdk4, p21, and p27) that causes cell cycle arrest at G1/S [5] and the induction of apoptosis by the phosphorylation of extracellular signal-regulated kinase (ERK) [7] have been reported.

Although most of the anticancer research on VK has focused on VK2 and VK3, there have also been studies demonstrating the VK1 anticancer effects [4]. The majority of the published data on VK1 are related to hepatocellular carcinoma (HCC) [6] and there are reports supporting the notion that also glioma and pancreatic cancer cell lines are sensitive to phylloquinone [7, 8]. In contrast, to our knowledge, no data are available on its antitumor effects on colon cancer cells.

Beyond the above cited mechanisms of cancer inhibition, a possible control by VK1 on different molecules involved in cell growth mechanisms could be hypothesized. In this context, the cellular polyamines spermidine (Spd) and spermine (Spm), as well as their precursor putrescine (Put), might be considered. These polycations are essential for growth and DNA synthesis and their intracellular content homeostasis is lost during dysregulation of cell proliferation, leading to cancer development [9]. Besides, the mucosal polyamine levels are known to be elevated in cancer cells compared to normal ones and they have also been suggested as specific markers for neoplastic proliferation [10]. Ornithine decarboxylase (ODC) is the key enzyme involved in polyamine biosynthesis. Increased ODC activity and the associated elevation in intracellular polyamines have been implicated in carcinogenesis of many human tissues, including large intestine [11]. Therefore, the polyamine biosynthesis can be regarded as an attractive target for cancer chemotherapy.

Different substances that affect polyamine metabolism, including ODC inhibitors and agents that stimulate polyamine catabolism, have been studied* in vitro* and* in vivo* as new potential therapeutic tools for cancer treatment and prevention [12]. But no data are available about the use of nutritional components such as VK1 for targeting the polyamine pathway in the treatment of CRC.

Starting from these bases, the aims of the present study were to investigate in three human colon cancer cells the effects of increasing concentrations of VK1 on (1) the cell proliferation, (2) the apoptotic processes with the possible involvement of the mitogen-activated protein kinase (MAPK) pathway, and finally (3) the polyamine biosynthesis. In order to evaluate if these effects could vary depending on the peculiarities of cancer cell lines, differently graded human colon cancer cell lines (Caco-2, HT-29, and SW480) were used.

## 2. Materials and Methods

### 2.1. Cell Culture Conditions

Human colon adenocarcinoma-derived Caco-2 cell line (well differentiated) (G1-2) (from adenocarcinoma), HT-29 cell line (moderately well differentiated) (G2) (from adenocarcinoma grade II), and SW480 cell line (low differentiated) (G3-4) (from adenocarcinoma grades III-IV) were obtained from the Interlab Cell Line Collection (Genoa, Italy). These colonic adenocarcinoma cells were used since they may represent the spectrum of cellular changes seen in precancerous lesions and manifest tumors [13]. Cells were routinely cultured in RPMI-1640, McCoy's 5A, and Leibovitz L-15 medium, respectively, supplemented with 10% fetal bovine serum (FBS), 2 mM glutamine, 100 U/mL penicillin, and 100 *μ*g/mL streptomycin, in a monolayer culture, and incubated at 37°C in a humidified atmosphere containing 5% CO_2_ in air. At confluence, the grown cells were harvested by means of trypsinization and serially subcultured with a 1 : 4 split ratio. All cell culture components were purchased from Sigma-Aldrich (Milan, Italy).

### 2.2. VK1 Treatment

Caco-2, HT-29, and SW480 cells (25th–30th passage) were seeded at a density of 2 × 10^5^ cells/5 mL of supplemented RPMI-1640, McCoy's 5A, and Leibovitz L-15 medium, respectively, in 60 mm tissue culture dishes (Corning Costar Co., Milan, Italy). After 24 h, to allow attachment the medium was removed and supplemented with culture medium containing increasing concentrations of VK1 (10 *μ*M, 50 *μ*M, 100 *μ*M, and 200 *μ*M) for 24 h, 48 h, and 72 h. Triplicate cultures were set up for each treatment and for the control, and each experiment was repeated 3 times. In the set of experiments investigating the role of ERK 1/2 in the VK1-induced apoptosis, the three cell lines were treated with 20 *μ*M MEK inhibitor (UO126) 2 h prior to 100 *μ*M and 200 *μ*M VK1 treatment for 48 h.

### 2.3. Assessment of Cell Proliferation

After Caco-2, HT-29, and SW480 cells had been cultured for 24 h, 48 h, and 72 h with increasing concentrations of VK1, the proliferative response was measured by colorimetric 3-(4,5 di-methylthiazol-2-yl)-2,5-diphenyltetrazolium bromide (MTT) test. To determine cell growth by colorimetric test, MTT stock solution (5 mg/mL in medium) was added to each dish at a volume of one-tenth the original culture volume and incubated for 2 h at 37°C in humidified CO_2_. At the end of the incubation period, the medium was removed, and the blue formazan crystals were solubilized with acidic isopropanol (0.1 N HCl absolute isopropanol). MTT conversion to formazan by metabolically viable cells was monitored by spectrophotometer at an optical density of 570 nm.

### 2.4. Apoptosis

The apoptosis was measured by evaluation of Bax and Bcl-2 mRNA expression (using quantitative PCR (qPCR) method with SYBR1 green dye) and protein expression of Bax, Bcl-2, caspase-3, and caspase-9 (using western blot analysis). Cells were washed twice in phosphate buffer saline (PBS) and then trypsinized and centrifuged at 280 ×g. The cell pellets were resuspended in 0.3 mL of pure distilled water and used for RNA extraction. Total cell RNA was extracted using Tri-Reagent (Mol. Res. Center Inc., Cincinnati, Ohio, USA), following the manufacturer's instructions. About 2 *μ*g total cell RNA, extracted from both the control and treated cells, was used for cDNA synthesis. Reverse transcription (RT) was carried out in 20 *μ*L of the final volume at 41°C for 60 min, using 30 pmol antisense primers for analyses of Bax, Bcl-2, and *β*-actin gene [14]. The *β*-actin gene was utilized as an internal control and was chosen as a reference gene because it is a housekeeping gene. Real-time PCRs were performed in 25 *μ*L of final volume containing 2 *μ*L of cDNA, master mix with SYBR Green (iQ SYBR Green Supermix Bio-Rad, Milan, Italy), and sense and antisense primers for Bax, Bcl-2, and *β*-actin gene. Real-time PCRs were carried out in a CFX96 Real-Time PCR Detection System (Bio-Rad Laboratories, Inc.) using the following protocol: 45 cycles at 95°C for 3 min, 95°C for 10 s, and 55°C for 30 s followed by a melting curve step at 65–95°C with a heating rate of 0.5°C per cycle for 80 cycles. The PCR products were quantified by external calibration curves, one for each tested gene, obtained with serial dilutions of known copy number of molecules (10^2^–10^7^ molecules). All expression data were normalized by dividing the target amount by the amount of *β*-actin used as internal control for each sample. The specificity of the PCR product was confirmed by gel electrophoresis.

As concerns western blot, Caco-2, HT-29, and SW480 cells were collected and lysed on ice in RIPA buffer (Pierce RIPA buffer, Thermo Scientific, Rockford, IL, USA). After homogenization and centrifugation at 18000 ×g for 15 min at 4°C, protein concentration was measured by a standard Bradford assay (Bio-Rad Laboratories, Milan, Italy). Aliquots of 50 *μ*g of total proteins were separated in 4–12% precast polyacrylamide gels (Invitrogen, Life Technologies, OR, USA) and transferred onto a PVDF membrane (Bio-Rad Laboratories, Milan, Italy) with the Transblot Turbo (Bio-Rad Laboratories). Bax, Bcl-2, caspase-3, caspase-9, P-ERK 1/2, and *β*-actin protein expressions were evaluated by 1 : 500 diluted Bax, Bcl-2 (D55G8), cleaved caspase-3 (Asp175), caspase-3 (3G2), cleaved caspase-9, caspase-9, phospho-p44/42 MAPK (ERK 1/2) (197G2), p44/42 MAPK (L34F12), and *β*-actin antibody, respectively (Cell Signaling Technology, Danvers, MA, USA). After overnight incubation, the membranes were further incubated with a horseradish peroxidase-conjugated goat secondary antibody (Bio-Rad Laboratories). The proteins were detected by chemiluminescence (ECL, Thermo Scientific, Rockford, IL, USA) and the densitometric analysis of each protein-related signal was obtained using the Molecular Imager Chemidoc (Bio-Rad Laboratories) and normalized against *β*-actin expression.

### 2.5. Polyamine Biosynthesis

For the evaluation of polyamine levels after VK1 treatment, each cell culture pellet was homogenized in 700 *μ*L of 0.9% sodium chloride mixed with 10 *μ*L (200 nmol/mL) of the internal standard 1,10-diaminodecane (1,10-DAD). An aliquot of the homogenate was used to measure the total protein content. Then, to precipitate proteins, 50 *μ*L of 3 M perchloride acid was added to the homogenate. After 30 min of incubation in ice, the homogenate was centrifuged for 15 min at 7000 ×g. The supernatant was filtered (Millex-HV13 pore size 0.45 *μ*m, Millipore, Bedford, MA, USA) and lyophilized. The residue was dissolved in 300 *μ*L of 0.1 N HCl. Dansylation and the extraction of dansyl-polyamine derivatives were performed as previously described [15]. After extraction, aliquots of 200 *μ*L were injected into a high-performance liquid chromatography system (UltiMate 3000, Dionex Corp., Sunnyvale, CA, USA) equipped with a reverse-phase column (Sunfire C18, 4.6 × 100 mm, 3.5 *μ*m particle size, Waters, Milford, MA, USA). Polyamines were eluted with a linear gradient ranging from acetonitrile-water (50 : 50, v : v) to acetonitrile (100%) for 30 min. The flow was 0.5–1.0 mL/min from 0 to 12 min and was then set at a constant rate (1.0 mL/min) until the 30th min. The fluorescent intensity was monitored by a fluorescence detector (UltiMate 3000 RS, Dionex Corp., Sunnyvale, CA, USA) with excitation at 320 nm and emission at 512 nm. Polyamine levels were expressed as concentration values in nmol/mg of protein.

As concerns ODC activity, it was measured with a radiometric technique that estimated the amount of ^14^CO_2_ liberated from DL-[1-^14^C]-ornithine (specific activity, 56.0 mCi/mmol; New England Nuclear) [16]. The cell culture pellet (2–4 × 10^6^ cells) was homogenized in 0.6 mL ice-cold 15 mM Tris-HCl (pH 7.5) containing 2.5 mM dithiothreitol, 40 *μ*M pyridoxal-5-phosphate, and 100 *μ*M ethylenediaminetetraacetate and then centrifuged at 30000 ×g for 30 min at 4°C. An aliquot of supernatant (200 *μ*L) was added to a glass test tube containing 0.05 *μ*Ci DL-[1-^14^C]-ornithine and 39 nmol DL-ornithine. After incubation for 60 min at 37°C, the reaction was stopped by adding trichloroacetic acid to a final concentration of 50%. ^14^CO_2_ liberated from DL-[1-^14^C]-ornithine was trapped on filter paper pretreated with 40 *μ*L of 2 N NaOH, which was suspended in a center well above the reaction mixture. Radioactivity on the filter papers was determined by a liquid scintillation counter (model 1219 Rackbeta; LKB-Pharmacia, Uppsala, Sweden). ODC activity was expressed as pmolCO_2_/h/mg of protein. Enzymatic activity was found to be linear within the range of 50–600 *μ*g of protein (*r* ^2^ = 0.99). The intra-assay and interassay variation coefficients (CV%) were 6% and 8%, respectively. The effects of VK1 treatment on ODC mRNA levels were evaluated using the above described qPCR method with SYBR1 green dye and the appropriate primers.

### 2.6. Statistical Analysis

Due to the nonnormal distribution of the data, nonparametric tests were performed. Data were analyzed by Kruskal-Wallis analysis of variance and Dunn's multiple comparison test. All data are expressed as mean and SEM. Differences were considered significant at *P* < 0.05. A specific software package (SigmaStat for Windows version 3.00 SPSS Inc., San Jose, CA, USA) was used.

## 3. Results

### 3.1. Effects of VK1 on Cell Proliferation

**Figure 1:**
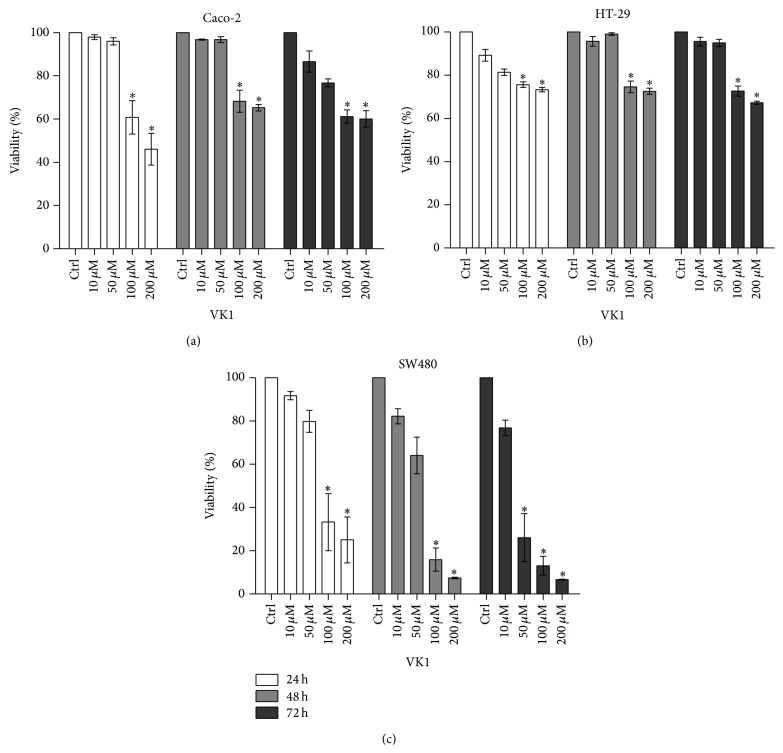
Proliferative response of Caco-2, HT-29, and SW480 cell lines to vitamin K1 (VK1) treatment. Effects of increasing concentrations of VK1 (10 *μ*M, 50 *μ*M, 100 *μ*M, and 200 *μ*M) on the conversion of MTT tetrazolium salt in Caco-2 (a), HT-29 (b), and SW480 (c) cell lines after 24 h, 48 h, and 72 h of treatment. All data represent the result of three different experiments (mean ± SEM). For each time of treatment, data were analyzed by Kruskal-Wallis analysis of variance and Dunn's multiple comparison test. ^*^ *P* < 0.05 compared to control cells.

Exposure of Caco-2, HT-29, and SW480 cell lines to increasing concentrations of VK1 up to 72 h caused an evident antiproliferative effect (Figure 1). After 24 h and 48 h, concentrations equal to or higher than 100 *μ*M caused a significant reduction (*P* < 0.05) in the conversion of the MTT tetrazolium salt in all the tested cells compared to untreated ones. After 72 h, the same concentrations remained effective for Caco-2 and HT-29 cells, while for SW480 a significant reduction (*P* < 0.05) was also caused by lower VK1 concentrations (starting from 50 *μ*M) compared to control cells. In addition, this cell line experienced a dramatic reduction in the percentage of viability at all the tested times with a decrease by approximately 90% at 200 *μ*M after 48 h and 72 h, thus demonstrating a more marked susceptibility to the antiproliferative action by VK1.

### 3.2. Effects of VK1 on Apoptosis

**Figure 2:**
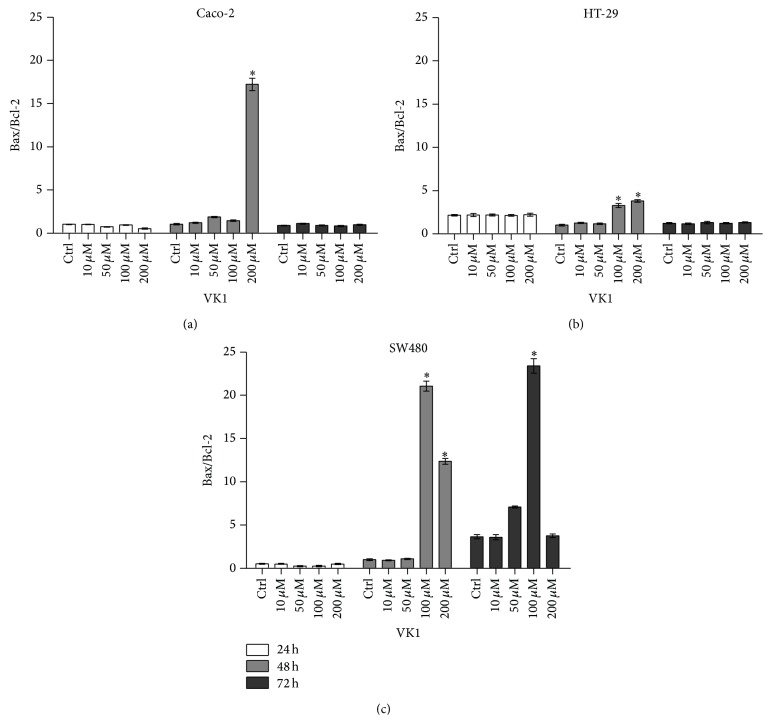
Apoptotic response of Caco-2, HT-29, and SW480 cell lines to vitamin K1 (VK1) treatment. Effects of increasing concentrations of VK1 (10 *μ*M, 50 *μ*M, 100 *μ*M, and 200 *μ*M) on the Bax/Bcl-2 mRNA level in Caco-2 (a), HT-29 (b), and SW480 (c) cell lines after 24 h, 48 h, and 72 h of treatment. All data represent the result of three different experiments (mean ± SEM). For each time of treatment, data were analyzed by Kruskal-Wallis analysis of variance and Dunn's multiple comparison test. ^*^ *P* < 0.05 compared to control cells.

Figure 2 shows the effects of increasing VK1 concentrations administered up to 72 h on apoptosis evaluated by Bax and Bcl-2 mRNA levels and expressed as Bax/Bcl-2 ratio. As concerns Caco-2 cells, a significant increase (*P* < 0.05) in the Bax/Bcl-2 ratio was observed only at 48 h and at the highest VK1 concentration (200 *μ*M) compared to control cells. At the same time, HT-29 cells exhibited a significant proapoptotic effect (*P* < 0.05) at VK1 concentrations equal to or higher than 100 *μ*M compared to untreated cells. As concerns SW480 cells, after 48 h of exposure the resulting Bax/Bcl-2 ratio significantly increased (*P* < 0.05) at VK1 concentrations equal to or higher than 100 *μ*M compared to control cells. Interestingly, after 72 h of treatment VK1 continued to significantly induce apoptosis only when administered at 100 *μ*M, while the Bax/Bcl-2 ratio dramatically decreased at the highest 200 *μ*M concentration, probably as a consequence of VK1 toxic effect causing an increase in the dead cell number.

**Figure 3:**
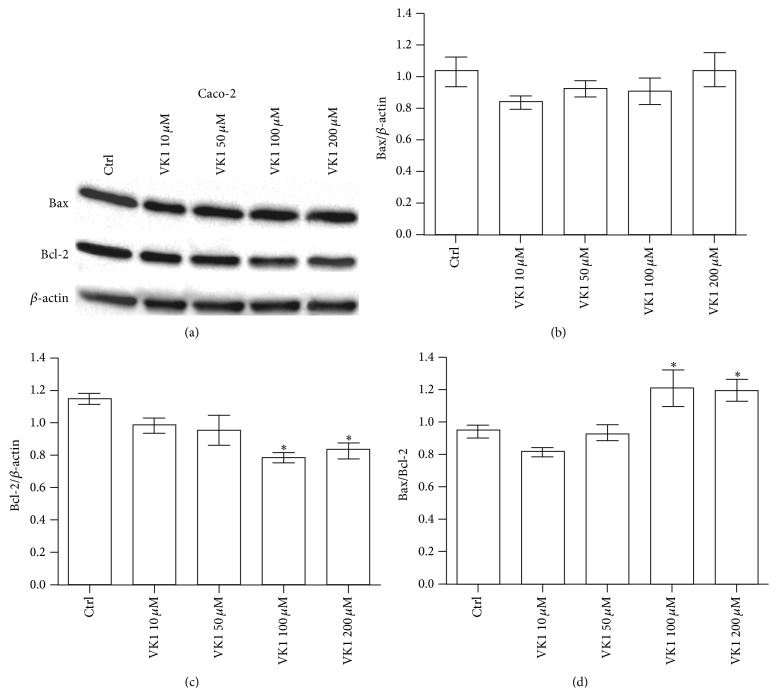
Western blot analysis of Bax and Bcl-2 in Caco-2 cells after 48 h of vitamin K1 (VK1) treatment. The cells were exposed to increasing concentrations of VK1 (10 *μ*M, 50 *μ*M, 100 *μ*M, and 200 *μ*M). Immunoreactive bands were quantified using Quantity One program. The diagrams show quantification of the intensity of bands, calibrated to the intensity of *β*-actin bands. All data represent the result of three different experiments (mean ± SEM). Data were analyzed by Kruskal-Wallis analysis of variance and Dunn's multiple comparison test. ^*^ *P* < 0.05 compared to control cells.

**Figure 4:**
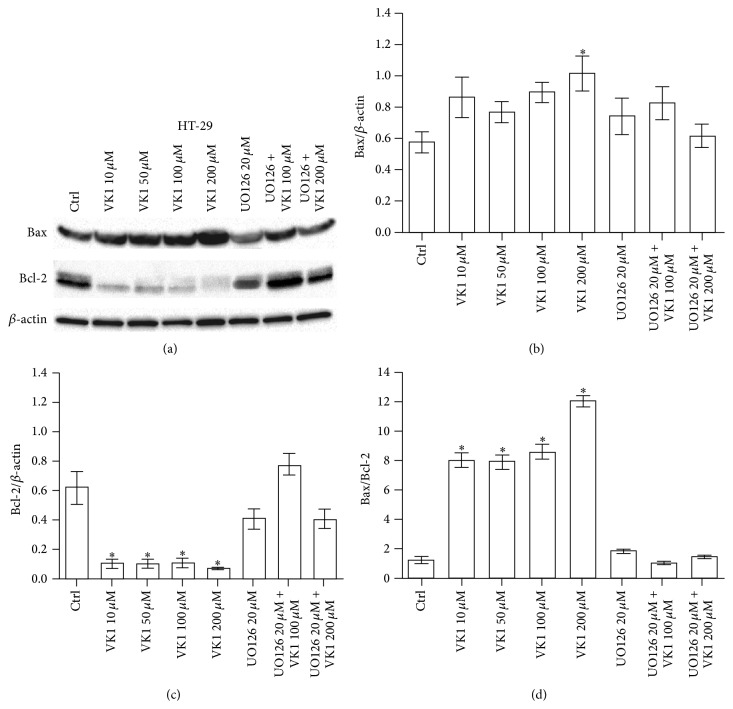
Western blot analysis of Bax and Bcl-2 in HT-29 cells after 48 h of vitamin K1 (VK1) treatment. The cells were exposed to increasing concentrations of VK1 (10 *μ*M, 50 *μ*M, 100 *μ*M, and 200 *μ*M), 20 *μ*M UO126 alone, or in combination with 100 *μ*M and 200 *μ*M VK1. Immunoreactive bands were quantified using Quantity One program. The diagrams show quantification of the intensity of bands, calibrated to the intensity of *β*-actin bands. All data represent the result of three different experiments (mean ± SEM). Data were analyzed by Kruskal-Wallis analysis of variance and Dunn's multiple comparison test. ^*^ *P* < 0.05 compared to control cells.

**Figure 5:**
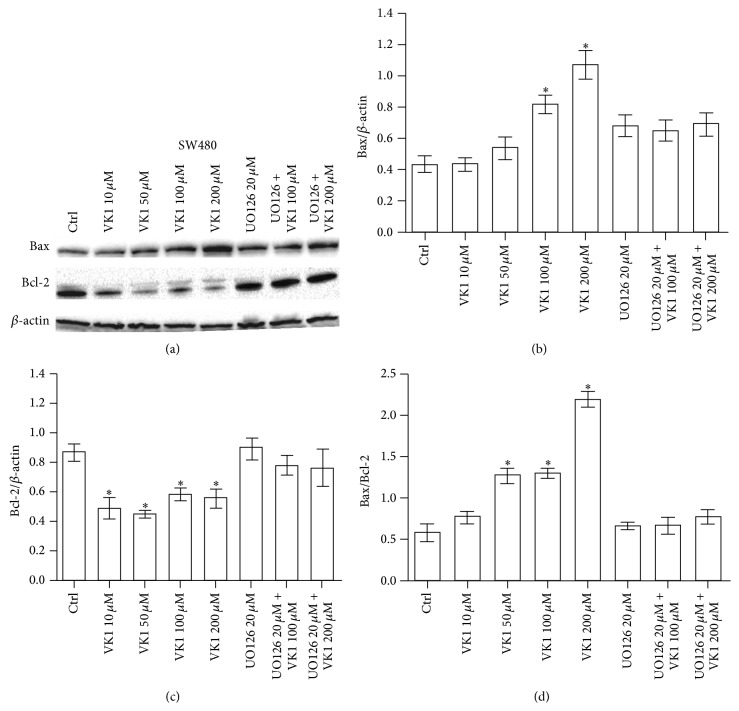
Western blot analysis of Bax and Bcl-2 in SW480 cells after 48 h of vitamin K1 (VK1) treatment. The cells were exposed to increasing concentrations of VK1 (10 *μ*M, 50 *μ*M, 100 *μ*M, and 200 *μ*M), 20 *μ*M UO126 alone, or in combination with 100 *μ*M and 200 *μ*M VK1. Immunoreactive bands were quantified using Quantity One program. The diagrams show quantification of the intensity of bands, calibrated to the intensity of *β*-actin bands. All data represent the result of three different experiments (mean ± SEM). Data were analyzed by Kruskal-Wallis analysis of variance and Dunn's multiple comparison test. ^*^ *P* < 0.05 compared to control cells.

**Figure 6:**
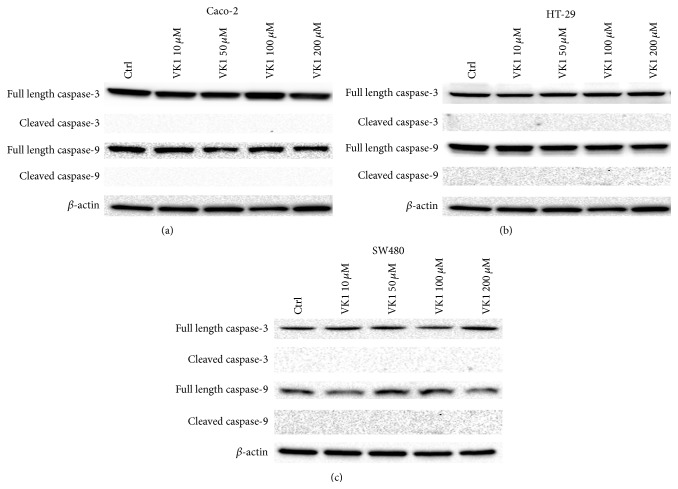
Western blot analysis of caspase-3 and caspase-9 in Caco-2, HT-29, and SW480 cells after 48 h of vitamin K1 (VK1) treatment. The cells were exposed to increasing concentrations of VK1 (10 *μ*M, 50 *μ*M, 100 *μ*M, and 200 *μ*M). Immunoreactive bands were quantified using Quantity One program.

The effects of increasing VK1 concentrations were also investigated by western blot analysis. The protein levels of Bax, Bcl-2, caspase-3, and caspase-9 were evaluated. A significant proapoptotic effect was observed only at 48 h. More specifically, Bax/Bcl-2 ratio in Caco-2 cells increased significantly (*P* < 0.05) at VK1 concentrations equal to or higher than 100 *μ*M, compared to control cells (Figure 3). This effect was caused only by a significant decrease (*P* < 0.05) of Bcl-2 protein compared to untreated cells. Besides, Bax/Bcl-2 ratio in HT-29 cells increased significantly (*P* < 0.05) at all the VK1 concentrations compared to control cells (Figure 4). In this cell line, Bax protein levels significantly increased (*P* < 0.05) at the highest VK1 concentration (200 *μ*M) while Bcl-2 decreased (*P* < 0.05) at all the VK1 concentrations compared to control cells. Finally, Bax/Bcl-2 ratio in SW480 cells increased significantly (*P* < 0.05) at VK1 concentrations starting from 50 *μ*M compared to untreated cells (Figure 5). Bax protein significantly increased (*P* < 0.05) at VK1 concentrations equal to or higher than 100 *μ*M, while Bcl-2 decreased (*P* < 0.05) at all the VK1 concentrations compared to control cells. The participation of caspases in VK1-induced apoptosis in the three cell lines was also evaluated. No significant cleavage of caspase-3, an executioner caspase, or caspase-9, an initiator caspase, after treatment with VK1 was observed (Figure 6). These findings suggest that VK1 is able to induce apoptosis in these colon cancer cells without involving the caspase activation.

### 3.3. Role of ERK 1/2 on the VK1-Induced Apoptosis

**Figure 7:**
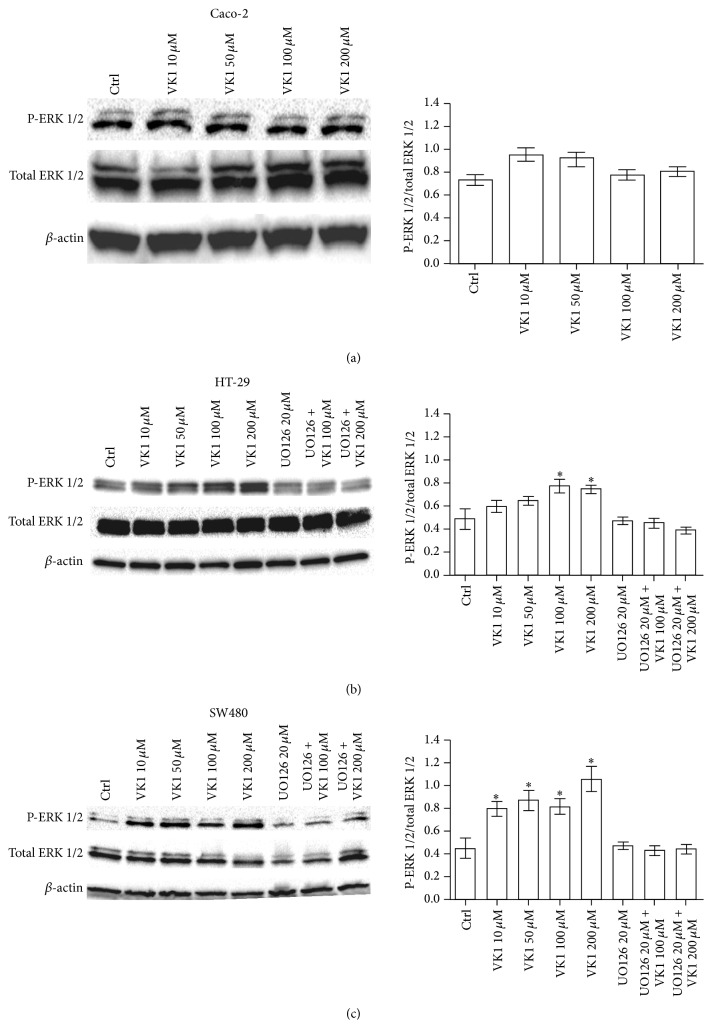
Western blot analysis of P-ERK 1/2 and total ERK 1/2 in Caco-2, HT-29, and SW480 cells after 48 h of vitamin K1 (VK1) treatment. The cells were exposed to increasing concentrations of VK1 (10 *μ*M, 50 *μ*M, 100 *μ*M, and 200 *μ*M) or to 20 *μ*M UO126 alone or in combination with 100 *μ*M and 200 *μ*M VK1. Immunoreactive bands were quantified using Quantity One program. The diagrams show quantification of the intensity of bands, calibrated to the intensity of *β*-actin bands. All data represent the result of three different experiments (mean ± SEM). Data were analyzed by Kruskal-Wallis analysis of variance and Dunn's multiple comparison test. ^*^ *P* < 0.05 compared to control cells.

Since ERK 1/2 has been linked to the regulation of cellular growth, apoptosis, and chemoresistance [17], the regulation of this signaling molecule by VK1 in colon cancer cells was investigated. The phosphorylation of ERK 1/2 was evaluated by treating cells with increasing concentrations of VK1 (from 10 *μ*M to 200 *μ*M) for 24 h, 48 h, and 72 h (data not shown). A dose-dependent increase in phospho-ERK 1/2 (P-ERK 1/2) was observed only after 48 h, in HT-29 and SW480 cells. The effect was significant (*P* < 0.05) at VK1 concentrations equal to or higher than 100 *μ*M in HT-29, while SW480 cells were susceptible to all the used VK1 concentrations (Figure 7). When the cells were pretreated with 20 *μ*M UO126 for 2 h, followed by VK1 treatment (100 *μ*M and 200 *μ*M) for 48 h, there was no induction of P-ERK 1/2 (Figure 7). Interestingly, the addition of the MEK inhibitor UO126 blocked sensitive colon cancer cells from VK1 mediated induction of apoptosis, thus indicating an involvement of the MAPK pathway in this process.

### 3.4. Effects of VK1 on Polyamine Biosynthesis

As concerns the polyamine profile, Table 1 shows the results obtained in Caco-2, HT-29, and SW480 cell lines following exposure to VK1 increasing concentrations up to 72 h. VK1 treatment led to a significant decrease of the single and total polyamine contents in all the tested cells. In detail, after 24 h the decrease in Caco-2 cells was significant (*P* < 0.05) compared to control cells as concerns Put, Spd, and the total polyamine content following exposure to VK1 concentrations equal to or higher than 100 *μ*M. After 48 h, lower VK1 concentrations (starting from 50 *μ*M) caused a significant reduction (*P* < 0.05) in Put and the total polyamine levels compared to untreated cells. After 72 h, the decrease was significant (*P* < 0.05) starting from the same VK1 concentration (50 *μ*M) for all the single and total polyamines. In contrast, in HT-29 cells the significant reduction (*P* < 0.05) in the single and total polyamine content was observed only after 48 h and 72 h of VK1 exposure. The decrease was significant (*P* < 0.05) at VK1 concentrations equal to or higher than 100 *μ*M, for all the single and total polyamines, compared to control cells. Finally, SW480 cells administered with VK1 concentrations equal to or higher than 100 *μ*M showed a significant reduction (*P* < 0.05) of the single and total polyamine contents compared to control cells at all the tested times.

**Figure 8:**
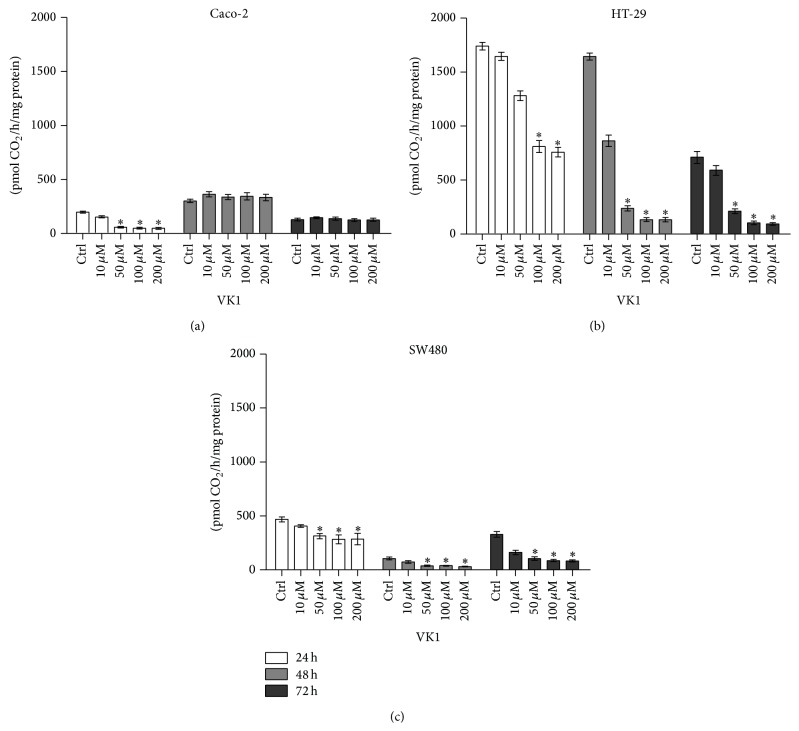
Ornithine decarboxylase (ODC) activity of Caco-2, HT-29, and SW480 cell lines in response to vitamin K1 (VK1) treatment. Effects of increasing concentrations of VK1 (10 *μ*M, 50 *μ*M, 100 *μ*M, and 200 *μ*M) on the ODC activity in Caco-2 (a), HT-29 (b), and SW480 (c) cell lines after 24 h, 48 h, and 72 h of treatment. All data represent the result of three different experiments (mean ± SEM). For each time of treatment, data were analyzed by Kruskal-Wallis analysis of variance and Dunn's multiple comparison test. ^*^ *P* < 0.05 compared to control cells.

ODC activity of Caco-2, HT-29, and SW480 cell lines following VK1 treatment was studied at increasing concentrations up to 72 h (Figure 8). As shown, ODC activity in Caco-2 cells was significantly reduced (*P* < 0.05) only after 24 h of exposure to VK1 concentrations equal to or higher than 50 *μ*M compared to untreated cells. In contrast, VK1 administered for 24 h at concentrations equal to or higher than 100 *μ*M to HT-29 cells significantly reduced (*P* < 0.05) ODC activity compared to control cells. In these cells, ODC activity was also significantly reduced by lower VK1 concentrations (starting from 50 *μ*M) administered for 48 h and 72 h. Finally, in the less differentiated SW480 cells, VK1 concentrations equal to or higher than 50 *μ*M significantly reduced (*P* < 0.05) ODC activity compared to untreated cells at all the tested times. As concerns ODC expression, no significant effect was observed in Caco-2 cells following VK1 treatment. In contrast, in HT-29 and SW480 cell lines, 24 h administration of VK1 concentrations equal to or higher than 100 *μ*M caused a significant reduction (*P* < 0.05) of ODC mRNA levels compared to untreated cells. Longer period of administration (48 h and 72 h) caused no significant effect on ODC mRNA levels (data not shown).

## 4. Discussion

VK, an essential nutrient often associated with the clotting cascade, has also been demonstrated to have an anticancer potential. Much of the available data focused on VK3, but also VK2 and VK1 have been shown to possess antineoplastic properties as reported in a variety of cancer cells including HCC, glioma, and pancreatic cell lines [6–8]. A number of hypotheses have been proposed suggesting for these compounds a possible role as inhibitors of the growth in different cancer cells by actively inducing cell cycle arrest, differentiation, and apoptosis [5, 6].

In the present study, the administration of increasing VK1 concentrations up to 72 h influenced the growth as well as the polyamine metabolism of three differently graded human colon cancer cell lines (namely, Caco-2, HT-29, and SW480). In particular, as demonstrated by the reduction in MTT conversion, all the tested cells showed a significant antiproliferative effect when exposed to the highest VK1 concentrations (100 *μ*M and 200 *μ*M). Interestingly, after 72 h a significant reduction in MTT conversion in SW480 was also caused by VK1 at 50 *μ*M, thus indicating a more pronounced susceptibility of this cell line to VK1 antiproliferative effect.

As concerns apoptosis, in our study the three cell lines exhibited a significant induction of apoptosis after 48 h of VK1 treatment, as indicated by the significant increase in the Bax/Bcl-2 ratio. In Caco-2 cells, the ratio increase was exclusively due to the significant downregulation of the antiapoptotic Bcl-2 levels. In contrast, a significant increase in the proapoptotic Bax protein levels was observed in the other two cell lines, although with different susceptibility to VK1 concentrations (200 *μ*M and 100 *μ*M for HT-29 and SW480, resp.). Other differences in Bax/Bcl-2 ratio were also present between HT-29 and SW480. As a matter of fact, SW480 cells showed a tenfold lower ratio compared to HT-29 ones. In the latter cell line, a more pronounced decrease of Bcl-2 protein occurred starting from the significant VK1 concentration of 10 *μ*M.

Overall, VK1 proved to be a potent stimulator of apoptosis in a manner similar to VK3 or VK2 analogs [18] and its effect was mediated mainly via Bcl-2 downregulation. The apoptotic process is determined by the balance of proapoptotic and antiapoptotic proteins and Bcl-2 represents a broad antiapoptotic factor and opposes cell deaths following ionizing radiation, cancer drugs, and hormonal manipulations [19].

Noteworthy, VK1-induced apoptosis was not mediated by the activation of caspases since western blot analysis revealed no active products of caspase-3, an executioner caspase, and caspase-9, an initiator caspase. Even if caspases are recognized players of apoptosis in various models, increasing evidence demonstrated that apoptosis may be induced without their activation [20].

To determine whether the proven VK1-induced cell death was associated with the activation of ERK, belonging to the MAPK family, western blot was performed. VK1 treatment caused a significant dose-dependent increase in ERK phosphorylation in a cell-type specific manner; HT-29 and SW480 cells exhibited a significant induction of P-ERK, starting from very different VK1 concentrations (100 *μ*M and 10 *μ*M for HT-29 and SW480, resp.). This induction was time-dependent with a peak increase after 48 h. Of note, simultaneous cotreatment of cells with a MEK inhibitor and VK1 at the highest concentrations (100 *μ*M and 200 *μ*M) prevented the induction of ERK phosphorylation. In addition, the MEK inhibitor blocked sensitive colon cancer cells from VK1-mediated induction of apoptosis, implicating the involvement of the MAPK pathway in this process.

The MAPK family, including ERK, the c-jun N-terminal kinase/stress-activated protein kinases (JNK/SAPK), and p38 kinases, has emerged as playing a crucial role in the cellular proliferation, differentiation, and apoptosis [21]. ERK pathway is known to prevent cell death, whereas the JNK/SAPK and p38 pathways have shown proapoptotic actions. However, reports in the literature have proposed a different interpretation of the phosphorylation of ERK. Leppä et al. [22] proved that ERK pathway stimulated c-jun synthesis and phosphorylation. Since c-jun itself is implicated in the apoptotic processes, the activation of the ERK pathway also can relate to cell death. Zhu et al. [23] showed a proapoptotic role of the ERK pathway in T-cell in which the inhibition of ERK phosphorylation antagonized apoptosis. Pumiglia et al. [24] provided evidence that a sustained increase of ERK activity inhibited cell cycle dependent kinase and induced growth arrest. Similar results were obtained more recently by Showalter et al. [8] in a study performed on pancreatic cancer cell lines treated with either VK1 or VK2 at inhibitory doses. Besides, Du et al. [7] demonstrated that VK1 enhanced the cytotoxicity effect of sorafenib through inhibiting the Raf/MEK/ERK signaling pathway in glioma cells. Matsumoto et al. [25] demonstrated that VK2 induced apoptosis and activated the MEK/ERK signaling pathway in liver cancer cells in a cell-type specific manner.

In order to identify other possible mechanisms implicated in VK1 ability to affect neoplastic cell growth, polyamines and their rate-limiting enzyme, ODC, were evaluated. A significant decrease in the polyamine content was observed at different VK1 concentrations (50 *μ*M for Caco-2 and 100 *μ*M for HT-29 and SW480 cells). Besides, after 24 h, a concomitant and significant decrease in ODC activity occurred in the three cell lines, starting from the concentration of 50 *μ*M. In HT-29 and SW480 cells this effect persisted also after 48 h and 72 h.

A significant decrease in ODC gene expression occurred only in HT-29 and SW480 cells after 24 h of VK1 treatment. In Caco-2 cell line the decrease of ODC activity after 24 h of treatment was not sustained by a concomitant inhibition of gene expression, probably due to the fact that VK1 exerts its effects on the modulation of protein enzymatic activity without any consequence on ODC gene.

ODC is a key regulator in polyamine metabolism, being now considered as a true oncogene [26]. This enzyme influences mainly Put and Spd levels, which are more involved in cell proliferation than Spm; the latter is implicated essentially in cell differentiation and neoplastic transformation, with different processes involved in maintaining its critical levels. High ODC expression along with increased polyamine concentrations is associated with fast proliferating cells [27]. In CRC, polyamine levels are significantly increased compared with either normal or preneoplastic tissue and are considered as reliable markers of neoplastic cell proliferation [28]. Polyamines stabilize chromatin and nuclear enzymes due to their ability to create complexes with organic polyanions, such as groups of proteins and DNA. It has been proposed that stabilization of the chromatin structure by polyamines may represent a mechanism by which these molecules affect nuclear processes including cell division and apoptosis [29]. Moreover, it is known that polyamines influence the expression of various genes involved in cell proliferation, tumor invasion, and metastasis [30].

To our knowledge, this is the first study investigating the behavior of polyamine metabolism on colon cancer cell lines treated with VK1 and a significant decrease in the polyamine content was demonstrated. A similar response has also been observed in previous studies performed in the same as well as other cell lines treated with drugs or natural molecules that inhibit polyamine biosynthesis, such as DFMO, anandamide, or probiotics [14, 31, 32].

As concerns the apoptotic processes, interestingly, an active involvement of either the MAPK pathway or the polyamine metabolism could be hypothesized, since a link between polyamine decrease and apoptosis exists [33]. It has been demonstrated that the depletion of polyamines can lead to cell cycle arrest or apoptosis by affecting numerous cell cycle regulatory pathways [34]. Polyamines have been proven as pivotal in regulating ion transport and stabilization of different cellular components such as cell membranes and chromatin structure. Hence, a decrease in the levels of these polycations might cause a destabilization of important cell structures, leading to alterations in cell integrity and inducing cell death.

We used three human colon cancer cells with distinct degree of differentiation, according to their grade of infiltration or metastasis, in an attempt to represent the variety of cellular changes occurring in precancerous lesions as well as manifest tumors. The obtained data suggest that the antiproliferative and proapoptotic effects by VK1 were also related to the characteristics of the colon cancer cell line. In fact, the less differentiated SW480 cells (from a moderately differentiated colon adenocarcinoma, grade IV Duke B) showed a more pronounced susceptibility to VK1 action due to a more marked antiproliferative effect, an increased Bax/Bcl-2 ratio persisting up to 72 h, and a significant increase in the phosphorylation of ERK 1/2 starting from low VK1 concentrations in comparison to the more differentiated HT-29 and Caco-2 cell lines. These data are in accordance with the reported high capacity of* de novo* polyamine biosynthesis and of polyamine uptake ascribed to this cell line, representing probably a precondition for the rapid growth ability shown by SW480 [35].

Regarding the different doses of VK1 used in this study, concentrations ranging from 10 *μ*M to 200 *μ*M can be considered pharmacological since normal serum levels of VK1 were found to be approximately 0.61 ng/mL. Yet, VK1 appears to be without toxicity and studies performed in humans established that mega doses of VK1 (up to 1000 mg orally daily) were used without side effects in patients with HCC [36]. Additionally, other studies clearly indicated that VK1 alone did not alter P-ERK levels, except at very high concentrations starting from 100 *μ*M, the same concentrations needed to exert the anticancer effects seen in our human colon cancer cells [37].

## 5. Conclusions

Undoubtedly, the significance of investigations performed* in vitro* related to the* in vivo* situations has to be taken with caution due to the presence of several differences; besides, cell sensitivity is only one factor and is not necessarily the most important in determining specificity of VK1 action. Anyway, it is possible to postulate that the colon cancer cells may be a good candidate tumor system to evaluate the antiproliferative and proapoptotic ability of VK1. Our future studies will be aimed at continuing to uncover the exact mechanisms underlying VK1 anticancer effect in colon cancer cells and whether polyamine depletion by itself is directly responsible for the observed proapoptotic action. Finally, considering that VK1 is safe and nontoxic, further research in humans is required to support these properties, also focusing better on the doses needed to obtain* in vivo* the same relevant effects produced in experimental models. From a therapeutic point of view, more probably, combinations of natural substances such as VK1 with other compounds (e.g., polyamine inhibitors and/or analogues) would enhance their properties, representing a suitable option for chemoprevention and/or treatment of colon cancer.

## Figures and Tables

**Table 1:** The polyamine profile: putrescine (Put), spermidine (Spd), spermine (Spm), and total polyamine (Total) in Caco-2, HT-29, and SW480 cell lines following administration of vitamin K1 (VK1) increasing concentrations (from 10 *μ*M to 200 *μ*M) after 24 h, 48 h, and 72 h.

Caco-2		Control	VK1 10 *μ*M	VK1 50 *μ*M	VK1 100 *μ*M	VK1 200 *μ*M
24 h	Put	0.70 ± 0.06	0.34 ± 0.03	0.30 ± 0.05	0.15 ± 0.03^∗^	0.13 ± 0.03^∗^
	Spd	12.17 ± 0.44	12.30 ± 0.95	9.43 ± 0.35	8.13 ± 1.07^∗^	7.93 ± 0.71^∗^
	Spm	11.63 ± 0.33	12.07 ± 0.52	10.87 ± 0.29	11.53 ± 0.26	11.33 ± 0.67
	Total	**24.50** ± **0.72**	**24.71** ± **0.39**	**20.60** ± **0.62**	19.82 ± 0.86^∗^	19.40 ± 0.70^∗^

48 h	Put	0.87 ± 0.07	0.57 ± 0.07	0.33 ± 0.04^∗^	0.32 ± 0.03^∗^	0.32 ± 0.05^∗^
	Spd	16.47 ± 0.33	10.53 ± 0.37	9.63 ± 0.38	8.87 ± 0.29^∗^	8.53 ± 0.29^∗^
	Spm	13.07 ± 0.69	12.73 ± 0.48	12.13 ± 0.47	12.60 ± 0.49	11.97 ± 0.022
	Total	**30.41** ± **0.83**	**23.57** ± **0.74**	21.96 ± 0.70^∗^	21.78 ± 0.39^∗^	21.15 ± 0.53^∗^

72 h	Put	0.57 ± 0.04	0.48 ± 0.05	0.19 ± 0.03^∗^	0.24 ± 0.03^∗^	0.23 ± 0.03^∗^
	Spd	10.53 ± 0.32	9.72 ± 0.40	6.51 ± 0.46^∗^	5.89 ± 0.31^∗^	5.93 ± 0.48^∗^
	Spm	15.83 ± 0.35	14.93 ± 0.46	13.25 ± 0.52^∗^	13.37 ± 0.43^∗^	13.40 ± 0.38^∗^
	Total	**26.94** ± **0.70**	**25.13** ± **0.92**	19.95 ± 0.88^∗^	19.50 ± 0.67^∗^	19.56 ± 0.75^∗^

HT-29		Control	VK1 10 *μ*M	VK1 50 *μ*M	VK1 100 *μ*M	VK1 200 *μ*M

24 h	Put	0.76 ± 0.05	0.65 ± 0.04	0.53 ± 0.13	0.55 ± 0.10	0.58 ± 0.08
	Spd	13.04 ± 0.05	12.67 ± 0.18	13.03 ± 0.12	12.93 ± 0.18	12.76 ± 0.23
	Spm	9.70 ± 0.32	9.93 ± 0.28	9.60 ± 0.63	10.13 ± 0.54	9.97 ± 0.24
	Total	**23.49** ± **0.39**	**23.27** ± **0.35**	**23.30** ± **0.76**	**23.71** ± **0.55**	**23.38** ± **0.47**

48 h	Put	1.41 ± 0.11	1.14 ± 0.23	1.06 ± 0.18	0.33 ± 0.12^∗^	0.22 ± 0.04^∗^
	Spd	10.11 ± 0.27	9.43 ± 0.24	9.45 ± 0.19	6.93 ± 0.49^∗^	6.25 ± 0.16^∗^
	Spm	14.40 ± 0.47	14.47 ± 0.20	13.40 ± 0.40	7.27 ± 0.39^∗^	7.13 ± 0.42^∗^
	Total	**25.92** ± **0.81**	**25.04** ± **0.55**	**23.91** ± **0.43**	14.53 ± 0.91^∗^	13.60 ± 0.54^∗^

72 h	Put	0.53 ± 0.18	0.52 ± 0.08	0.30 ± 0.03	0.27 ± 0.04^∗^	0.21 ± 0.04^∗^
	Spd	8.44 ± 0.29	8.46 ± 0.31	7.50 ± 0.43	7.23 ± 0.34^∗^	6.60 ± 0.32^∗^
	Spm	13.50 ± 0.32	10.50 ± 0.46	8.30 ± 0.32	6.87 ± 0.38^∗^	6.87 ± 0.97^∗^
	Total	**22.47** ± **0.79**	**19.48** ± **0.84**	**16.10** ± **0.66**	14.37 ± 0.76^∗^	13.68 ± 1.29^∗^

SW480		Control	VK1 10 *μ*M	VK1 50 *μ*M	VK1 100 *μ*M	VK1 200 *μ*M

24 h	Put	2.24 ± 0.20	1.45 ± 0.09	1.03 ± 0.14	0.72 ± 0.12^∗^	0.78 ± 0.15^∗^
	Spd	13.27 ± 0.26	11.74 ± 0.14	9.73 ± 0.32	8.53 ± 0.41^∗^	8.24 ± 0.33^∗^
	Spm	12.93 ± 0.29	12.03 ± 0.20	10.77 ± 0.29	9.33 ± 0.18^∗^	9.22 ± 0.23^∗^
	Total	**28.44** ± **0.75**	**25.22** ± **0.43**	**21.53** ± **0.75**	18.59 ± 0.64^∗^	18.24 ± 0.50^∗^

48 h	Put	1.59 ± 0.17	1.04 ± 0.18	0.79 ± 0.11	0.35 ± 0.06^∗^	0.36 ± 0.09^∗^
	Spd	9.93 ± 0.29	8.73 ± 0.39	7.60 ± 0.40	6.02 ± 0.36^∗^	5.83 ± 0.45^∗^
	Spm	13.13 ± 0.40	13.05 ± 0.62	12.16 ± 0.20	11.68 ± 0.29^∗^	11.87 ± 0.24^∗^
	Total	**24.65** ± **0.52**	**22.82** ± **0.45**	**20.55** ± **0.72**	18.05 ± 0.53^∗^	18.06 ± 0.54^∗^

72 h	Put	1.79 ± 0.11	1.57 ± 0.14	1.65 ± 0.09	0.93 ± 0.12^∗^	0.93 ± 0.13^∗^
	Spd	7.31 ± 0.21	6.58 ± 0.25	6.27 ± 0.17	4.91 ± 0.27^∗^	4.77 ± 0.22^∗^
	Spm	12.58 ± 0.23	12.18 ± 0.18	12.38 ± 0.38	12.03 ± 0.16^∗^	11.83 ± 0.20^∗^
	Total	**21.68** ± **0.55**	**20.33** ± **0.46**	**20.30** ± **0.64**	17.87 ± 0.44^∗^	17.53 ± 0.47^∗^

^∗^ *P* < 0.05 compared to control cells.

## References

[1] Lepage C., Hamza S., Faivre J. (2010). Epidemiologie et recommandations pour le depistage du cancer colorectal [Epidemiology and screening of colon cancer]. *La Revue du Praticien*.

[2] Russo A., Corsale S., Cammareri P. (2005). Pharmacogenomics in colorectal carcinomas: future perspectives in personalized therapy. *Journal of Cellular Physiology*.

[3] Shearer M. J., Newman P. (2008). Metabolism and cell biology of vitamin K. *Journal of Thrombosis and Haemostasis*.

[4] Lamson D. W., Plaza S. M. (2003). The anticancer effects of vitamin K. *Alternative Medicine Review*.

[5] Ozaki I., Zhang H., Mizuta T. (2007). Menatetrenone, a vitamin K2 analogue, inhibits hepatocellular carcinoma cell growth by suppressing cyclin D1 expression through inhibition of nuclear factor *κ*B activation. *Clinical Cancer Research*.

[6] Mizuta T., Ozaki I. (2008). Hepatocellular carcinoma and vitamin K. *Vitamins and Hormones*.

[7] Du W., Zhou J.-R., Wang D.-L., Gong K., Zhang Q.-J. (2012). Vitamin K1 enhances sorafenib-induced growth inhibition and apoptosis of human malignant glioma cells by blocking the Raf/MEK/ERK pathway. *World Journal of Surgical Oncology*.

[8] Showalter S. L., Wang Z., Costantino C. L. (2010). Naturally occurring K vitamins inhibit pancreatic cancer cell survival through a caspase-dependent pathway. *Journal of Gastroenterology and Hepatology*.

[9] Moinard C., Cynober L., de Bandt J.-P. (2005). Polyamines: metabolism and implications in human diseases. *Clinical Nutrition*.

[10] Thomas T., Thomas T. J. (2003). Polyamine metabolism and cancer. *Journal of Cellular and Molecular Medicine*.

[11] Shantz L. M., Levin V. A. (2007). Regulation of ornithine decarboxylase during oncogenic transformation: mechanisms and therapeutic potential. *Amino Acids*.

[12] Casero R. A., Marton L. J. (2007). Targeting polyamine metabolism and function in cancer and other hyperproliferative diseases. *Nature Reviews Drug Discovery*.

[13] Brunner H., Hausmann F., Krieg R. C. (2001). The effects of 5-aminolevulinic acid esters on protoporphyrin IX production in human adenocarcinoma cell lines. *Photochemistry and Photobiology*.

[14] Linsalata M., Notarnicola M., Tutino V. (2010). Effects of anandamide on polyamine levels and cell growth in human colon cancer cells. *Anticancer Research*.

[15] Linsalata M., Russo F., Notarnicola M., Berloco P., Di Leo A. (1998). Polyamine profile in human gastric mucosa infected by *Helicobacter pylori*. *Italian Journal of Gastroenterology and Hepatology*.

[16] Garewal H. S., Sloan D., Sampliner R. E., Fennerty B. (1992). Ornithine decarboxylase assay in human colorectal mucosa. Methodologic issues of importance to quality control. *International Journal of Cancer*.

[17] McCubrey J. A., Steelman L. S., Chappell W. H. (2007). Roles of the Raf/MEK/ERK pathway in cell growth, malignant transformation and drug resistance. *Biochimica et Biophysica Acta: Molecular Cell Research*.

[18] Wang Z., Wang M., Finn F., Carr B. I. (1995). The growth inhibitory effects of vitamins K and their actions on gene expression. *Hepatology*.

[19] Sentman C. L., Shutter J. R., Hockenbery D., Kanagawa O., Korsmeyer S. J. (1991). bcl-2 inhibits multiple forms of apoptosis but not negative selection in thymocytes. *Cell*.

[20] Mareninova O. A., Sung K.-F., Hong P. (2006). Cell death in pancreatitis: caspases protect from necrotizing pancreatitis. *The Journal of Biological Chemistry*.

[21] Sui X., Kong N., Ye L. (2014). P38 and JNK MAPK pathways control the balance of apoptosis and autophagy in response to chemotherapeutic agents. *Cancer Letters*.

[22] Leppä S., Saffrich R., Ansorge W., Bohmann D. (1998). Differential regulation of c-Jun by ERK and JNK during PC12 cell differentiation. *The EMBO Journal*.

[23] Zhu L., Yu X., Akatsuka Y., Cooper J. A., Anasetti C. (1999). Role of mitogen-activated protein kinases in activation-induced apoptosis of T cells. *Immunology*.

[24] Pumiglia K. M., Pumiglia K. M., Decker S. J., Decker S. J. (1997). Cell cycle arrest mediated by the MEK/mitogen-activated protein kinase pathway. *Proceedings of the National Academy of Sciences of the United States of America*.

[25] Matsumoto K., Okano J.-I., Nagahara T., Murawaki Y. (2006). Apoptosis of liver cancer cells by vitamin K2 and enhancement by MEK inhibition. *International Journal of Oncology*.

[26] Pegg A. E. (2006). Regulation of ornithine decarboxylase. *Journal of Biological Chemistry*.

[27] Alm K., Oredsson S. (2009). Cells and polyamines do it cyclically. *Essays in Biochemistry*.

[28] Linsalata M., Caruso M. G., Leo S., Guerra V., D'Attoma B., Di Leo A. (2002). Prognostic value of tissue polyamine levels in human colorectal carcinoma. *Anticancer Research*.

[29] Thomas T., Thomas T. J. (2001). Polyamines in cell growth and cell death: molecular mechanisms and therapeutic applications. *Cellular and Molecular Life Sciences*.

[30] Paz E. A., Garcia-Huidobro J., Ignatenko N. A. (2011). Polyamines in cancer. *Advances in Clinical Chemistry*.

[31] Wallace H. M., Fraser A. V. (2004). Inhibitors of polyamine metabolism: review article. *Amino Acids*.

[32] Linsalata M., Russo F. (2008). Nutritional factors and polyamine metabolism in colorectal cancer. *Nutrition*.

[33] Seiler N., Raul F. (2005). Polyamines and apoptosis. *Journal of Cellular and Molecular Medicine*.

[34] Igarashi K., Kashiwagi K. (2010). Modulation of cellular function by polyamines. *International Journal of Biochemistry and Cell Biology*.

[35] Duranton B., Holl V., Schneider Y. (2003). Polyamine metabolism in primary human colon adenocarcinoma cells (SW480) and their lymph node metastatic derivatives (SW620). *Amino Acids*.

[36] Wei G., Wang M., Hyslop T., Wang Z., Carr B. I. (2010). Vitamin K enhancement of sorafenib-mediated HCC cell growth inhibition in vitro and in vivo. *International Journal of Cancer*.

[37] Wei G., Wang M., Carr B. I. (2010). Sorafenib combined vitamin K induces apoptosis in human pancreatic cancer cell lines through RAF/MEK/ERK and c-Jun NH2-terminal kinase pathways. *Journal of Cellular Physiology*.

